# Beyond PSA: The Future of Prostate Cancer Diagnosis Using Artificial Intelligence, Novel Biomarkers, and Advanced Imagery

**DOI:** 10.3390/life15101508

**Published:** 2025-09-25

**Authors:** Moncef Al Barajraji, Mathieu Coscarella, Ilyas Svistakov, Helena Flôres Soares da Silva, Paula Mata Déniz, María Jesús Marugan, Claudia González-Santander, Lorena Fernández Montarroso, Isabel Galante, Juan Gómez Rivas, Jesús Moreno Sierra

**Affiliations:** 1 Department of Urology, Hôpital Universitaire de Bruxelles, Université Libre Bruxelles, 1070 Brussels, Belgium; moncef.al.barajraji@ulb.be (M.A.B.); mathieucoscarella@gmail.com (M.C.); ilyas.svistakov@humani.be (I.S.); 2 Department of Urology, Hospital de Clínicas de Porto Alegre, Universidade Federal do Rio Grande do Sul, Porto Alegre 91509-900, Brazil; helena.margot0403@gmail.com; 3 Department of Urology, Instituto de Investigación Sanitaria, Hospital Clínico San Carlos, 28040 Madrid, Spain; paulamatadeniz@gmail.com (P.M.D.); chusi.marugan@gmail.com (M.J.M.); claudiaglezsantander@gmail.com (C.G.-S.); lorenafmonty@gmail.com (L.F.M.); m.isabel.galante@gmail.com (I.G.); jemoreno@ucm.es (J.M.S.); 4 Department of Urology, Universidad Complutense de Madrid, 28015 Madrid, Spain

**Keywords:** prostate cancer, PSA, artificial intelligence, MRI, multimodal diagnosis

## Abstract

Prostate cancer (PCa) diagnosis has historically relied on the prostate-specific antigen (PSA) testing. Although the screening significantly reduces mortality rates, PSA has low specificity with risks of overdiagnosis and overtreatment. These limitations highlight the need for a more accurate diagnostic approach. Emerging technologies, such as artificial intelligence (AI), novel biomarkers, and advanced imaging techniques, offer promising avenues to enhance the accuracy and efficiency of PCa diagnosis and risk stratification. This narrative review comprehensively analyzed the current literature, focusing on new tools aiding PCa diagnosis (AI-driven image interpretation, radiomics, genomic classifiers, biomarkers, and multimodal data integration) with consideration for technical, regulatory, and ethical challenges related to clinical implementation of AI-based technologies. A literature search was performed using the PubMed and MEDLINE databases to identify relevant peer-reviewed articles published in English using the search terms “prostate cancer,” “artificial intelligence,” “machine learning,” “deep learning,” “MRI,” “histopathology,” and “diagnosis.” Articles were selected based on their relevance to AI-assisted diagnostic tools, clinical utility, and performance metrics. In addition, a separate section was developed initially to contextualize the limitations of current PSA-based screening approaches. The reviewed studies showed that AI had significant utility in prostate mpMRI interpretation (lesion detection; Gleason grading) with high accuracy and high reproducibility. For the pathologist, AI-driven algorithms improve the diagnostic accuracy of digital slide evaluation for histologic diagnosis of prostate cancer and automated Gleason score grading. Genomic tools such as the Oncotype DX test, combined with AI, could also allow for tailored and individualized risk prediction. Overall, multimodal models integrating clinical, imaging, and molecular data often outperform traditional PSA-based strategies and reduce unnecessary biopsies. Transition from PSA-centered toward AI-driven, biomarker-supported, and image-enhanced diagnosis marks a critical evolution in PCa diagnosis.

## 1. Introduction

Nowadays, thinking about prostate cancer (PCa) screening, diagnosis, and prognosis without the prostate-specific antigen (PSA) is almost impossible. Until PSA’s isolation in 1979 by Dr. Ming Chu’s research group [1,2] and, later, the development of an ELISA immunoassay that could be used for its blood detection [3], one of the only biomarkers available to evaluate PCa, the prostatic acid phosphatase, had the limitation of only becoming elevated in men with metastatic bone disease [4]. From these early PSA studies, it was not believed that PSA could be used as a screening tool for PCa; until the early 1990s, it was primarily used for monitoring treatment response in patients who already had an established PCa diagnosis [2,5], and it was already known that patients with other prostatic diseases, such as benign prostatic hyperplasia (BPH) and prostatitis, could also present higher PSA levels. The use of PSA as a first-line screening blood test came mainly from Catalona’s 1991 [6,7] and 1994 [7] trials, which compared digital rectal examination with PSA testing to detect PCa early and led to its approval as an aid to PCa early detection by the United States Food and Drug Administration (FDA). From this time on, a new era started, marked by widespread PSA testing with an initial decline of 45–70% in PCa mortality due to this screening by the year 2000 [8]. 

However, widespread screening for PCa using PSA blood rapidly began to raise concerns considering its diagnostic limitations. In a recent meta-analysis over PSA diagnostic accuracy for detection of PCa among clinically referred men, the consensual biopsy threshold of PSA (≥4.0 ng/ml) demonstrated 93% sensitivity for early detection of PCa but with only 20% specificity [9]. This low specificity is due to the fact that PSA is also increased by benign prostate conditions (e.g., BPH and prostatitis) and poses a risk of unnecessary biopsies to rule out eventual malignancy [9,10]. The other downside of widespread PSA screening is the risk of overtreatment associated with overdiagnosis. PCa is heterogeneous; it can vary from indolent to aggressive; and patients can be diagnosed with PCa without benefits in survival from this detection and even experience side effects related to interventions, monitoring, and treatment of the disease. Several trials with extended follow-up periods, such as the Stockholm3 and ProtecT trials, have also demonstrated the benefits of taking repeated PSA dosages between 3 and 10 ng/mL when there is a clinically uncertain suspicion of PCa to avoid these unnecessary biopsies, which is now part of current guidelines [10]. However, a risk of missing clinically significant PCa (Gleason > 6) persists considering the interindividual and intra-tumoral heterogeneity of PCa features. Furthermore, alternative medicine and complementary therapies are becoming popular among patients, especially in Western countries, illustrating patients’ desire for more personalized health management with less aggressive tools [11]. In this context, there is an urgent need for more precise and specific diagnostic tools incorporating the latest technologies available in the biomedical field, such as artificial intelligence (AI), novel biomarkers, and advanced imagery. This comprehensive review explores emerging diagnostic trends integrating these advanced technologies into the PCa diagnostic pathway, emphasizing the future of PCa diagnosis.

## 2. Limitations of PSA Screening for PCa Diagnosis

In some cases, PCa can be found in biopsy cores even at normal levels [10]. It is also known that increases in PCa detection rates are proportional to increases in PSA’s thresholds when performing a biopsy [12], with a common cutoff of 4 ng/mL consensually decided upon in the 1990s as a balance between sensitivity and specificity [13], although the risk of PCa exists for all PSA values [14]. Multiple studies have evaluated the sensitivity and specificity of PSA for diagnosing PCa, yielding variable results with the influence of factors such as age, intrinsic population characteristics, and the presence of PCa symptoms before testing. Twenty years ago, the Prostate Cancer Prevention Trial study tried to establish a PSA value cutoff point for the detection of PCa with simultaneously high sensitivity and high specificity among 18,882 healthy men of 55 years or more without PCa and with serum PSA ≤ 3.0 ng/mL and normal digital rectal examination [14]. The patients were followed up over 7 years with annual PSA measurement and digital rectal examination. Performing prostate biopsies was recommended if PSA exceeded 4.0 ng/mL or the patient underwent an abnormal digital rectal examination. After 7 years, an end-of-study prostate biopsy was recommended in all cancer-free patients. When considering PSA cutoff values of 1.1, 2.1, 3.1, and 4.1 ng/mL for the detection of PCa from any Gleason grade, the sensitivities were, respectively, 83.4%, 52.6%, 32.2%, and 20.5%, whereas specificities were, respectively, 38.9%, 72.5%, 86.7%, and 93.8%. With a cutoff of 1.1 ng/mL, the PCa detection rate increased to 83.4% but exposed 61.1% of men without PCa to a prostate biopsy. In comparison, at this time, a PSA threshold of 4.1 ng/mL had a sensitivity of only 20.5% for PCa detection but a specificity of 93.8%. The authors also noted that PSA sensitivity tended to increase among men with Gleason ≥ 8 PCa, with a sensitivity of 50.9% at 4.1 ng/mL [14]. However, they concluded that no cutoff value was reliable and that a continuum of prostate cancer risk existed through all values of PSA [14]. 

In this scenario of doubt between the pros and cons of lowering or increasing PSA cutoff levels to indicate prostate biopsy, the risks of unnecessary biopsies and overtreatment should also be considered. Although biopsy techniques have improved over time, the procedure remains associated with potential complications (pain, hematuria, infection, and rectal bleeding) with a non-negligible rate of false-negative results [15,16,17,18]. Besides the risks of biopsy, potential overdiagnosis and overtreatment of not clinically significant biopsy-proven tumors of low grade (Gleason 6) or low volume pose a problem. It is now accepted that low-grade and low-volume tumors should be actively surveilled and treated only with evidence of growth or change in aggressivity to avoid overtreatment while preserving low rates of metastasis and cancer-specific mortality (CSM) [19,20]. Data from the CAPSURE PCa registry, a longitudinal observational study of approximately 15,000 men with all stages of biopsy-proven PCa, showed, for example, that 92–98% of patients who had the lowest tumor scores and were potentially eligible for active surveillance were aggressively treated by radical surgery, radiation therapy, or hormone therapy [20]. This means that at least 92% of men who had low tumor scores with a 10-year CSM of 2.8% received aggressive treatment [20]. Quality of life impairment from overtreatment should also be considered when evaluating the effects of a screening program. Analysis of the European Randomized Study of Screening for Prostate Cancer (ERSPC) [21] concluded that the benefit of PSA screening was altered by loss of quality-adjusted life-years (QALYs) from the post-diagnosis long-term effects of PCa treatment modalities, such as sexual dysfunction, poor urinary function, and altered bowel function [22,23,24]. If overdiagnosis and overtreatment induce risks for patients, and missing early diagnosis of aggressive PCa implies higher mortality, then distinguishing indolent and aggressive PCa remains an important challenge, and surrogate factors are needed alongside PSA values to evaluate the risk of PCa and its aggressivity and select patients who should not undergo prostate biopsy and/or aggressive management without missing those who should. To define tumor aggressivity, current epidemiologic studies frequently use a combination of TNM staging, Gleason grade, and PSA values at diagnosis, mainly because these data are systematically available in large population-based studies. However, the heterogeneity of outcome definitions across these studies limits the possibility of comparing and combining results into meta-analyses. Hence, although we have achieved a better understanding of the differences between indolent and aggressive forms of PCa, it is still impossible to accurately predict PCa behavior, which can lead to treating indolent tumors aggressively or undertreating aggressive tumors. In this context of uncertainty and a lack of homogeneous definition, efforts have been made over the years to develop better diagnostic and management pathways with AI-based tools.

## 3. AI and Machine Learning in Prostate Cancer Diagnosis

AI is the ability of machines and software to perform tasks that would usually require human intellect to be performed. Machine learning (ML) is a domain of AI focused on developing models and algorithms that can improve their performance by exposing and identifying patterns in data over time without having been explicitly programmed for every act or decision. Considering that, AI and ML can be applied to diagnostic purposes in many situations. Considering that ML is powerful in image recognition and image pattern identification, two research fields where it has been primarily evaluated are radiology—and more specifically, prostate MRI interpretation—and pathology diagnosis. 

### 3.1. AI in Imaging

Multiparametric Magnetic Resonance Imaging (mpMRI) of the prostate integrates functional and anatomical MRI sequences, including T1- and T2-weighted images, diffusion-weighted images (DWIs), apparent diffusion coefficient (ADC) maps, and dynamic contrast-enhanced (DCE) images. In recent large studies, the specificity of prostate mpMRI for the detection of csPCa in biopsy-naïve patients ranged from 84% to 92% [25,26]. A recent meta-analysis with the same focus on csPCa lesions detection in prostate mpMPRI in biopsy-naïve patients further reported a positive predictive value of less than 40% but with an up to 90.8% negative predictive value [27]. This implies that despite the high accuracy of mpMRI for the detection of csPCa lesions, diagnosis of these lesions may be missed in at least 10% of patients if they do not undergo prostate biopsies. Prostate mpMRI could also be interesting for prognostic evaluation by using functional sequences to predict tumor behavior [27]. However, variations in image acquisition methods and protocols across institutions can result in variations in imaging quality that impair all image comparisons. Furthermore, MRI interpretation requires a steep learning curve while still being subject to variability in inter-observer interpretation [28]. In this context, AI and ML have been implicated in recent years in prostate mpMRI interpretation to allow for more accurate image reading for higher detection rates for PCa lesions, as well as allowing for volume estimation and lesion characterization. This computer-assisted interpretation increases the sensitivity and specificity of PCa detection in complement with radiologists [27,28,29,30]. 

When considering PCa imaging, one of the most important goals is prostate segmentation, which consists of identifying and delineating the boundaries of the gland in images to accurately separate it from surrounding tissues and identify the prostate capsule and any eventual capsular deformation or effraction. Prostate segmentation has multiple applications, from cancer diagnosis and surgical planning to prostate fusion biopsy and brachytherapy [29]. Segmentation of the prostate by an AI-driven process on prostate mpMRI images is a main research focus in the field of AI. The PROMISE12 dataset challenge is an example of this research. In that study, automated and semi-automated algorithms were applied by various institutions taking part in this challenge to a dataset of 100 prostate mpMRI studies from five different institutions. Overall, AI-based automated algorithms achieved the best results in prostate segmentation compared to other algorithms [30]. In terms of prostate volume estimation, which is a measure of high value for surgical planning of radical prostatectomy and PCa prognosis, prostate mpMRI assessment by AI-based algorithms also achieved good results in evaluating volume estimation and prostate segmentation according to several studies [31,32,33]. 

Furthermore, AI can also aid in lesion identification, tumoral volume estimation, and lesion segmentation. Before 2022, one of the biggest challenges for these processes was creating a fully automated system, and before then, all regions of interest (ROIs) on prostate mpMRI images had to be manually located beforehand after having trained the deep learning (DL) model [32]. In 2022, a model simultaneously performing fully automated detection, segmentation, and Gleason grade estimation on prostate mpMRI images was developed with promising initial results [33]. A decade earlier, the European Society of Urogenital Radiology introduced, in 2012, the Prostate Imaging Reporting and Data System (PI-RADS), an interpretation algorithm to standardize the acquisition, interpretation, and reporting of prostate mpMRI findings [34]. Subsequent updates led to the latest version, PI-RADS v2.1, in 2019 [34]. PI-RADS v2.1 evaluates several imaging sequences from prostate mpMRI to score the findings into a risk category ranging from 1 to 5. The likelihood of significant PCa increases as the PI-RADS v2.1 score increases. However, the score relies on mpMRI interpretation with widely recognized problems regarding heterogeneity among image interpretation by radiologists due to variable experience and variable times required to interpret these images. In this situation, ML techniques offer solutions for accurate and faster interpretation, and several studies have shown that AI algorithms could provide a PI-RADS score for prostate lesions, even outperforming experienced radiologists [35,36]. 

Radiomics is another important concept of AI. Radiomics consists of extracting features from radiographic computational images to characterize disease patterns. In PCa diagnosis, use of radiomics has been associated with encouraging results for PCa risk stratification [35], prediction of Gleason scores [36], and prediction of treatment outcomes [37]. For example, a recent multi-center study demonstrated the role of peri-tumoral radiomics associated with heterogeneity patterns around the tumor for the prediction of prostate cancer risk in bi-parametric MRI studies [35]. In another large recent study by Telecan et al., the association of clinical data with radiomics features extracted from prostate mpMRI T2-weighted sequence images showed high accuracy (>90%) in predicting prostate lesion aggressivity [38]. Table 1 provides a short overview of recent studies on the use of AI for PCa identification on prostate MRI.

### 3.2. AI in Pathology

Digital pathology involves converting traditional glass microscope slides into high-resolution digital images using specialized scanning devices. These whole-slide images (WSIs), digitally converted from the original slides, are particularly useful for the application of AI algorithms to identify areas of concern, confirm initial diagnoses, and enhance diagnostic accuracy. Further, AI algorithms can also provide second opinions to evaluate the conclusions of pathologists [41,42,43,44,45,46,47]. The DeepGleason© system, shared in 2024, is one example of an AI-enhanced pathology analysis tool [41]. DeepGleason© uses a deep neural network model to automatically grade prostate cancer via digital pathology slides, enhancing the consistency and accuracy of Gleason grading by providing second opinions to pathologists. This tool, which was trained on 34,264 samples from 369 slides for Automated Gleason Grading of PCa, achieved very high accuracy (97.4%) and outperformed other methods for separating benign and malignant lesions (94% sensitivity, 98% specificity) and for differentiation between Gleason 3 and Gleason 4–5 lesions (91% sensitivity; 75% specificity). 

In a recent meta-analysis on AI diagnostic accuracy for PCa identification and Gleason grading in a histologic material evaluation of 24 studies (~8000 biopsies; 458 prostatectomies), Morozov et al. reported a sensitivity of 87–100% and specificity of 68–99% for AI Gleason grading of PCa lesions on pathology [42]. Other studies also reported the benefit of AI evaluation to evaluate the prognostic of patients diagnosed with prostate cancer [43,44,45,46,47]. In another recent meta-analysis by Marletta et al. also, the excellent results of AI for PCa detection and grading were confirmed while also noticing a correlation between AI-identified histologic features and prognostic factors of PCa, such as biochemical recurrence, extraprostatic extension, perineural invasion, and disease-free survival [47]. Table 2 provides an overview of some recent studies on AI use for PCa histopathological evaluation.

### 3.3. AI in Risk Prediction Models

Another relevant topic is the integration of AI into clinical data and genomic profiling, which enables more precise risk stratification and personalized management of PCa. Multi-omics consists of integrating data from different omics layers (e.g., transcriptomics, genomics, and proteomics) to better understand biological systems [48]. One way this integration can work is by employing AI algorithms to develop novel biomarkers from multi-omics using a combination of clinical data, digital pathology, and genomic data. Studies that used AI models to analyze genomic and clinical data could predict which patients with PCa are more likely to benefit from certain treatments [49]. 

AI tools also have the power to automate the processing and analysis of huge genomic datasets, which could allow for the incorporation of genomic profiling into everyday clinical care. For example, the Oncotype DX assay [50] evaluated the use of prostate biopsy cores to predict tumor aggressiveness by measuring the RNA expression of PCa-related genes and generating a Genomic Prostate Score (GPS) of increased risk for worse pathology (Gleason 4–5, non-confined disease). However, biomarkers such as Oncotype Dx^®^ have not been FDA approved considering that additional data with comparisons to other parameters, such as MRI, are required before these markers can be routinely used in daily practice [10]. Another recent study evaluated an AI prediction model for passive screening of patients for PCa by analyzing clinical data from electronic medical records to identify high-risk patients. The model achieved equivalent sensitivity to PSA screening but with a 38% increase in specificity [51].

## 4. Integrating AI, Biomarkers, and Imaging for a Unified Diagnostic Model

**Figure 1:**
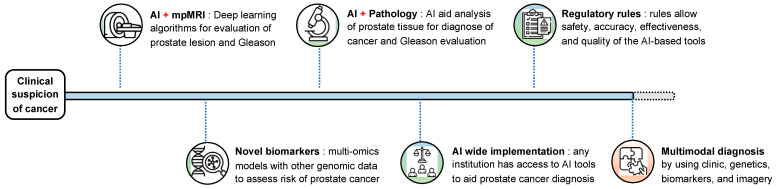
Evolution of prostate cancer diagnosis toward multimodal diagnosis.

“Multimodal precision diagnosis” in PCa utilizes AI to integrate multiple data sources, including genetic and molecular biomarkers, imaging, and clinical evidence, to provide accurate and personalized diagnosis and risk assessment of the disease, as illustrated by Figure 1. Many studies have been conducted in this regard, with results showing that multimodal AI (integrating deep learning lesion suspicion levels, PSA, prostate volume, patient age, and MRI-based features) can outperform clinical or MRI-only AI evaluation for the detection of clinically significant PCa [51,52]. Multimodal tools also improve risk stratification of suspect lesions, reducing the risk of unnecessary biopsies [51]. However, these models face challenges in full implementation within daily urologist practices. Factors such as significant costs to acquire personalized AI software and ensure its maintenance over time, the need for resources to convert conventional pathology slides into fully digital images, and training physicians for these new technologies are among the limitations to consider when aiming to implement a new AI multimodal system, especially in non-tertiary care institutions [52]. 

Another barrier to widespread use of AI in PCa diagnostics is the need to standardize diagnostic methods, as MRI image acquisition and interpretation are the areas that suffer the most from a lack of standardization across the published studies. It is mandatory that all institutions and researchers use the same format for digital mpMRI images and equivalent/reproducible software packages for analysis because the heterogeneity in currently available data limits the possibility of comparing results between multiple centers. To achieve a universal application of AI in clinical settings, it is also imperative to have regulatory measures for this new technology. AI technologies should be required to adhere to regulatory standards regarding safety, accuracy, and effectiveness, not only for quality control but also for consistent progress. 

Another important aspect is the need for transparency in how AI works for its users so that it is accessible and user-friendly for everyday practitioners, developers, and those who will inspect and regulate it. It is also important to note that AI technology providers should be required to be accountable for their actions through the application of supervision by healthcare providers and by having all parties involved in the development and deployment of AI to prevent eventual errors and harm to individuals. In this context, some regulatory organs have already introduced actions to better address the risks of inadvertent use of AI, such as the Artificial Intelligence Act (AIA) by the European Commission [53].

## 5. Future Perspectives and Clinical Implementation

Considering the expansion of AI models within the medical field, it is certain that these tools can potentially revolutionize daily urological practice by improving PCa diagnosis accuracy, treatment planification, and lowering unnecessary biopsies for better patient outcomes. However, it is essential to note that AI use in clinical practice should not be exempt from ethical obligations. The primary ethical dilemmas associated with AI in clinical settings include the risk of violating patients’ privacy, compromising their autonomy, and exposing them to potential biases. Data privacy should be guaranteed systematically through specific protocols for de-identification and transmission or sharing of the data. In terms of autonomy, patients have the right to participate in the decision-making process of their treatment and to receive full information about the risks and benefits of the management options. Indeed, medical actions based on AI algorithms can lead to the loss of the shared decision-making process between the patient and care provider without the patient’s fully informed consent over the treatment [54]. 

There are also still doubts about how the rights and privacy of patient data will be ensured, given the absence of universally applied regulatory measures and a lack of definition of who or which entities are responsible for potential errors and violations. Another limitation is the attribution of the responsibility for a potential error. A clear definition of liability in cases of AI-related diagnostic error should be built with human cross-control protocols. Considering both the pros and cons of the widespread use of AI tools within daily urology practice, future research on this topic must focus on large-scale validation studies and real-world implementation of this technology to reduce biases from AI algorithms being “trained” only on data from a specific demographic or on poorly standardized images. It is crucial that AI algorithms are trained on diverse datasets and frequently reviewed to ensure any biases and/or inequalities will not persist [55]. 

Other findings from cost-effectiveness analyses and workflow integration studies could also assist in the practical implementation of these tools. A huge future perspective on the use of AI in urology is its implementation in PCa screening programs over the next few decades. One of its main uses will reflect the shift to more sophisticated and personalized screening approaches by having AI-driven risk prediction models that integrate clinical data (age; family history), PSA trends, molecular analysis, genetic data, guided biopsies, and imaging (mpMRI and histopathological findings) in the process. But this widespread implementation first requires validation, and future research on prostate cancer focusing on AI-driven decision support tools must address critical key points around the accuracy, effectiveness, and applicability of these technologies. This involves conducting extensive validation trials to ensure AI can be effectively implemented in real-world settings with evaluations of long-term outcomes in various clinical environments.

## 6. Conclusions

PCa diagnosis is undergoing a paradigm shift from PSA-centered approaches to approaches based on AI, novel biomarkers, and advanced imagery. These innovations promise greater precision, improved risk stratification, and less overtreatment. However, widespread adoption of AI tools requires multidisciplinary collaboration, regulation, and equitable implementation for all. As we move forward, multimodal diagnostic models will be essential to providing personalized and data-driven care for patients at risk for PCa.

## Figures and Tables

**Table 1:** AI in prostate cancer imagery.

Study	Objectives	Main Findings
Arif, M. et al. (2020) [32]	csPCa detection and segmentation in 292 low-risk patients by a convolutional neural network model on prostate MRI	The model achieved a high sensitivity (82–92%) with a variable specificity (43–76%) based on lesion volume (AUC of 0.65–0.89)
Pellicer-Valero, O et al. (2022) [33]	Testing a fully automated DL model for csPCa detection, lesion segmentation, and Gleason grade evaluation on MRI	High sensitivity and specificity for prostate lesion segmentation (100% and 79%) and for PCa detection (100% and 80%) on MRI
Algohary, A. et al. (2020) [35]	Evaluation of combining peri-tumoral and intra-tumoral radiomics of prostate MRI images for risk stratification	Radiomics using the peri-tumoral and intra-tumoral features had accuracy of 53% (vs. 48% PI-RADS) for PCa risk stratification
Zhuang, H. et al. (2023) [36]	Evaluation of radiomics features for Gleason estimation using enlarged ROIs in 26 biopsy-proven PCa patients	Radiomics achieved 73.96% accuracy (Gleason ≥ 3+4 vs. 3+3) vs. 83.72% (Gleason 3+4 vs. ≥4+3) for radiologist-drawn ROIs
Bayerl, N. et al. (2024) [39]	Assessment of a fully automated diagnostic AI software for prostate MRI evaluation and pathological correlation	AI software showed 100% sensitivity for PI-RADS ≥ 2 lesions and 85.5% sensitivity with 63.2% specificity for PI-RADS ≥ 4 lesions
Saha, A. et al. (2024) [40]	International large study with 10,207 studies over 10 years comparing AI and radiologists for csPCa detection on MRI	In 400 cases, AI outperformed 62 radiologists for PCa detection

AI, artificial intelligence; AUC, area under the curve; csPCa, clinically significant prostate cancer; DL, deep learning; ML, machine learning; MRI, magnetic resonance imagery; PCa, prostate cancer; PI-RADS, prostate imaging reporting and data system; ROIs: regions of interest.

**Table 2:** AI in prostate cancer pathology.

Study	Objectives	Results
Steiner, D. F. et al. (2020) [43]	Evaluation of merging AI and pathologist evaluation to review and grade prostate biopsy cores in 240 patients	AI reviews showed a 5.6% improvement in agreement with pathologist (69.7% to 75.3%; *p* < 0.001). AI reviews also improved tumoral detection, time needed for review, self-confidence, and pathologists’ inter-agreement
Spratt, D. E. et al. (2022) [44]	Evaluation of an AI-powered digital pathology-based biomarker to predict ADT results in localized PCa with validation in NRG/RTOG 9408 studies with 1719 patients	AI-derived ADT biomarker evaluation showed benefit in the ADT group (HR 0.62, *p* = 0.006). In the biomarker-positive subgroup (39%), the ADT improved outcomes (HR 0.33, *p* < 0.001). Median follow-up of 17.4 years
Morozov, A. et al. (2023) [42]	Meta-analysis of AI diagnostic accuracy in diagnosing PCa and evaluating Gleason grades based on 24 studies with 8000 biopsies and 458 prostatectomy specimens	AI had high sensitivity (87–100%) and high specificity (68–99%) for PCa diagnosis. Meta-analysis pooled sensitivity of 0.96 and specificity of 0.95
Müller, D. et al. (2024) [41]	Evaluating a neural network AI system (DeepGleason©) trained on 34,264 tiles from 369 slides for automated Gleason grading of PCa on pathologic prostate samples	High accuracy (97.4%) with outperformance of conventional methods to separate benign from malignant lesions (sensitivity 94%, specificity 98%) and Gleason 3 from Gleason 4–5 lesions (sensitivity 91%, specificity 75%)

ADT, androgen deprivation therapy; AI, artificial intelligence; AUC, area under the curve; HR, hazard ratio; PCa, prostate cancer.

## Data Availability

No new data were created or analyzed in this study. Data sharing is not applicable to this article.
